# Preventing HIV transmission among Iranian prisoners: Initial support for providing education on the benefits of harm reduction practices

**DOI:** 10.1186/1477-7517-5-21

**Published:** 2008-06-09

**Authors:** Babak Eshrati, Rahim Taghizadeh Asl, Colleen Anne Dell, Parviz Afshar, Peggy Margaret E Millson, Mohammad Kamali, John Weekes

**Affiliations:** 1 Arak University of Medical Science, Arak, Iran; 2 United Nations Development Programme, Tehran, Iran; 3 Department of Sociology, University of Saskatchewan, Saskatchewan, Canada; 4 Health and Correction Deputy of Prison Organization, Tehran, Iran; 5 Department of Public Health Sciences, University of Toronto, Toronto, Canada; 6 Iran Medical University, Tehran, Iran; 7 Institute of Criminology and Criminal Justice, Carleton University, Ottawa, Canada

## Abstract

**Background:**

Harm reduction is a health-centred approach that seeks to reduce the health and social harms associated with high-risk behaviors, such as illicit drug use. The objective of this study is to determine the association between the beliefs of a group of adult, male prisoners in Iran about the transmission of HIV and their high-risk practices while in prison.

**Methods:**

A cross-sectional study was conducted in 2004. The study population was a random selection of 100 men incarcerated at Rajaei-Shahr prison. The data were collected through a self-administered questionnaire. Focus group discussions were held at the prison to guide the design of the questionnaire. The relationship between components of the Health Belief Model (HBM) and prisoners' risky HIV-related behaviors was examined.

**Results:**

Calculating Pearson's correlation coefficient, a significant, positive association was found between the benefit component of the HBM and prisoners *not *engaging in HIV high-risk behaviors.

**Conclusion:**

Educational harm reduction initiatives that promote the effectiveness of strategies designed to reduce the risk of HIV transmission may decrease prisoners' high-risk behaviors. This finding provides initial support for the Iran prison system's current offering of HIV/AIDS harm reduction programming and suggests the need to offer increased education about the effectiveness of HIV prevention practices.

## Background

Injection drug use and high-risk sexual behaviors are key contributing factors to the transmission of the human immunodeficiency virus (HIV). These behaviours have been identified in international research as two of the most common modes of HIV transmission in the prison setting [1,2]. They have also been identified as main contributing factors to increasing rates of HIV infection in Iran generally [3]. The opium ban in Iran has led to greater heroin use and injecting, and hence elevated rates of HIV infection through the sharing of injection equipment [4]. Alongside this, the government's focus on illicit drug supply-reduction has resulted in the prison becoming progressively more populated with individuals serving sentences for drug-related crimes and using drugs. HIV transmission in Iranian prisons has become a major concern for the country [5].

Harm reduction is a health-centered approach that seeks to reduce the health and social harms associated with high-risk behaviors [6,7]. Harm reduction initiatives are commonly targeted toward specific high-risk populations, including prisoners. A key component of such initiatives is taking a non-judgemental approach to the choices individuals make (e.g., decreased use of illicit drugs). There are various, although intermittent, forms of HIV-related harm reduction programs available to injection drug users within the Iranian prison system, ranging from methadone maintenance therapy to the provision of sterile equipment to inject drugs. Likewise, harm reduction initiatives such as condom distribution exist for individuals at risk of HIV due to their sexual practices. Although the contemporary Iranian prison system response to HIV transmission among its prisoner population has been very progressive, there remains considerable room for improvement [3,8].

In order to increase harm reduction programming across the Iranian prison system, the effectiveness of its application needs to be empirically established. The objective of this study is to determine the association between the beliefs of a group of adult, male prisoners in Iran about the transmission of HIV/AIDS and their high-risk practices while in prison. We begin by establishing the beliefs prisoners' hold about how HIV is transmitted. We then identify the types of behaviours prisoners engage in that have the potential for HIV transmission. Using the Health Belief Model (HBM) framework, and calculating statistical measures such as Pearson's correlation coefficient, we examine the association between prisoners' beliefs and practices. Based on the findings, it is suggested that educational harm reduction initiatives that promote the effectiveness of strategies designed to reduce the risk of HIV transmission may decrease prisoners' high-risk behaviors.

The Health Belief Model (HBM) focuses on the attitudes and beliefs of individuals and attempts to explain and predict their health behaviors [9,10]. The HBM is a widely used framework to help explain health related behaviors, including sexual risk taking and the transmission of HIV/AIDS [11]. The HBM is comprised of three key components: threat (believed susceptibility to and severity of a health condition), benefits (believed effectiveness of strategies designed to reduce the risk or seriousness of impact of a health condition), and barriers (believed negative consequences that may result from taking particular health actions because of a health condition). Stated simply, a person is believed to take part in preventative health related behavior (e.g., use a condom) if they feel the negative health condition can be avoided, if they feel their particular action can avoid the negative health condition, and if they are able to put the recommended health action into practice. Within a harm reduction context, the HBM provides a systematic framework for examining the reasoning behind an individual's choice to decrease, maintain or increase their high-risk behaviour. This is important in a prison context as educating individuals about the health risks of their behaviors through training and counselling is a widely-supported form of health promotion and disease prevention.

## Methods

A cross-sectional study design was undertaken to determine the beliefs and associated high-risk behaviors connected with the transmission of HIV among a group of adult males incarcerated in Rajaei-Shahr prison. This maximum-security prison is located in Karaj city, which is approximately 70 km North West of Tehran, the capital city of Iran. The study sample is 100 adult males who were incarcerated in March, 2004. The total incarcerated population at the time was approximately 3,200 males and 300 females. The participants in our study were randomly selected from a roster prepared by the prison authorities. This roster was developed based on an existing list of incarcerated cases provided by the prison authority and who were deemed accessible (i.e., not in solitary confinement or have specific reservations associated with them). Our sample is representative of the majority of male prisoners incarcerated at the prison at the time of the study. At the time, the design did not allow us to consider the types of crimes individuals were jailed for. It can be stated though that the Iran Prisons Organization tends to incarcerate like individuals together (e.g., type of crime).

Participation in the study was voluntary and required verbal informed consent, with the guarantee of anonymity. The collected data is securely maintained by the researchers. The questionnaire consisted of 75 items and was available in Farsi (Persian). For those prisoners with low literacy levels, a designated and trained health staff member was available to read the questions out loud without influence on the confidential responses. The response rate was 100%, and all questionnaire data were completed. This high rate is explained in part by the support prisoners have for the health programs offered by the prison, as well as the opportunity to engage in an activity that is outside their regular routines. There was no incentive (e.g., gift) for participating. This study was part of a larger research project conducted between 2003 and 2005; this study examined the impact of harm reduction interventions in prison. The study was funded by the World Health Organization (WHO) and ethics was granted from the WHO – Special Programme for Research and Training in Tropical Diseases, and the Iran Prisons Organization ethics committee.

Among the 100 randomly selected participants who took part in the study, the mean age was 32.06 (*SD *= 8.54, *Range *= 20–53 years). The mean duration of incarceration was 5.30 years (*SD *= 5.14, *Range *= 1–17 years) and the median was 3.0 years. Nearly 47% of the sample reported illicit drug use during their lifetime, including while incarcerated. This is not surprising given that drug related offences comprise a large proportion of Iran's prison population. We did not ask specifically about drug use while incarcerated at Rajaei-Shahr prison because such a direct question could elicit mistrust and fear in the initial stage of a multi-part study.

Data were collected using self-report questionnaires. In order to design a valid, culturally competent and standardized questionnaire, we drew upon the results of our review of the literature, and held focus group discussions with 3 groups of prisoners, each consisting of 8–10 participants in Rajaei-Shahr prison in January, 2004. The participants were selected with the assistance of the prison health staff and key prisoners who knew individuals that would be interested, cooperative and represented varied reasons for their incarceration and belief systems. All focus group participants were informed about the objective of the discussion and their confidentiality was guaranteed. All focus group discussions were held in Farsi (Persian). Policy regarding confidentiality and anonymity in research at the prison prevented us from determining whether the participants in the focus groups also participated in completing a self-report questionnaire. Drawing on the results of the focus group discussions and literature review, a bank of questions was designed for the larger study, of which 75 were selected for our survey. Using the HBM as a framework, we classified prisoners' beliefs about the risk of HIV transmission as a consequence of various behaviours.

A limitation of our research methodology is that our use of a cross-sectional design did not allow us to identify any causal relationship due to the lack of time sequence confirmation between the cause and the outcome [12]. That is, we were not able to confirm the temporal sequence of the prisoners' beliefs and behaviours. Further, given the particularly challenging nature of the context of this study (e.g., cultural and social factors), it is difficult to make generalizations from our study to other settings.

## Results

### Beliefs about modes for HIV transmission

The majority of participants in this study have considerable knowledge about modes of HIV transmission. On average, 79.5% of responses to 14 suggested modes of HIV transmission were answered correctly. Awareness was greatest for sharing a razor (1 incorrect, 1 do not know) and needle sharing (1 incorrect, 1 do not know). However, 95 of the 100 prisoners incorrectly reported that shaking hands and kissing with an HIV infected individual can cause them to be infected. See Table 1.

**Table 1:** Prisoner beliefs about modes of HIV transmission

	Incorrect answer	Do not known	Correct answer
Males having sex with males	5	5	90
Males having sex with females	18	13	69
Insect sting	21	14	65
Louse sting	10	14	76
Scabies	12	10	78
Eating with shared dishes	17	8	75
Shaking hands and kissing	95	0	5
Blood transfusion	3	3	94
Sharing a razor	1	0	99
Needle sharing for drug use	1	1	98
Tattooing with shared needle	4	0	96
Dentistry with infected instruments	12	2	86
Barber with infected tools	13	2	85
Hejamat (venesection and cupping)	1	2	97

### Applying the Health Belief Model

Considering the three components of the HBM (perceived threats [susceptibility and severity], benefits, barriers), Cronbach's alpha was calculated for every question combination, and it was consistently greater than 70%. From here, a single variable for each component of the HBM was calculated through the summation of questions specific to each. However, as there were only 2 questions for perceived severity, each was considered separately.

The prevalence of responses to various preventive and high-risk behaviors for HIV transmission is shown in Table 2. In order to combine the HBM with the results of the behavior questions, we calculated a Cronbach's alpha of 77.4%. All 9 questions documenting the prevalence of HIV high-risk behaviours were summed to achieve a common score for each respondent showing high scores with respect to safer behaviours for the prevention of HIV transmission. The mean, standard deviation, minimum and maximum possible achievable score for each component of the HBM other than perceived severity are shown in Table 3.

**Table 2:** Reported prevalence of HIV high-risk behaviours in prison

	**Always**	**Some times**	**Never**
Using a condom when having sex	29	51	20
Needle sharing	6	23	71
Having sex with a male	1	20	79
Having extra-marital sex (with a female)	7	58	35
Tattooing with shared needles	4	37	59
Having history of Hejamat (cupping, venesection)	6	3	91
Victim of a sexual assault	3	4	93
Being raped by other prisoners	2	66	32
Using a shared razor	37	52	11

**Table 3:** Statistical measures for 3 components of the Health Belief Model

	Mean	SD	Minimum	Maximum	Maximum Score
Benefits	11.39	3.81	7	22	32
Barriers	14	4.71	6	24	24
Susceptibility	12	3.5	7	25	28

Considering the least-harmful behaviour for each question (always, sometimes, never) as a correct answer, we associated a score and then calculated a total behavior score for each respondent. The mean for the correct behavior score was 22.29 (*SD *= 2.89, Min = 13, Max = 27). The maximum behavior score was 27. The calculated Pearson's correlation coefficient and the related *p *value for the three components of the HBM and associated behaviors showed a significant positive correlation *only *between the benefit component of the HBM and behavior (*r *= .29, *p *< .003). It should be noted that this is a weak to moderate range correlation.

In order to determine the association of behaviors with perceived severity we performed an analysis of variance with regard to the two questions. There was no statistically significant association between these two variables (*p *> .05).

## Discussion

The majority of prisoners in our study were knowledgeable about how HIV is transmitted. Their high level of understanding may be due in large part to recent credible HIV training efforts in Iranian prisons. The need and positive impact of training on awareness of HIV transmission has been documented in other international studies [13-15]. However, the vast majority of prisoners in our study still believed that HIV could be transmitted through kissing or hand shaking. This is consistent with a study conducted with prisoners in Nigeria [16]. A study of Iranian high school students in Tehran similarly found that the majority of respondents answered knowledge questions about HIV/AIDS correctly, but that there still existed misconceptions about the routes of transmission [17]. So, even with recent awareness training at Rajaei-Shahr prison, there is evidence of some inaccurate information about HIV transmission among the prisoner population.

Using the Health Belief Model (HBM) as the framework to help understand individuals' health related behaviors, specifically high-risk behaviors for the transmission of HIV, our results show that the only component of the model significantly associated with the reduction of high-risk behavior is perceived benefit. That is, prisoners decreased their HIV high-risk behaviours (e.g., used clean syringes) when they believed in the effectiveness of strategies designed to reduce the risk or seriousness of impact of the health condition. This does not mean that the other two components of the HBM are not effective in explaining health related behaviour, only that they did not show to be for the prisoner population in our study. Clearly, further research is required.

Similar to the findings in our study, a 2006 comparative study conducted in six cities in Eastern Europe, Asia and Latin America found that the promotion of and advocacy surrounding the health benefits of needle exchange for injection drug users positively affected HIV high-risk taking behaviors [18]. Another 2006 study, this one focussing on the feasibility of offering late-night harm reduction services for a hard to reach group of Methamphetamine-using men who have sex with men, concluded that providing needle exchange, condoms, sexually transmitted infection testing and harm reduction education *together *may positively impact the high-risk behaviors of individuals at risk for acquiring or transmitting HIV [19]. In other studies it has been shown that relaying the benefits of harm reduction strategies, as conceived in a HBM framework, may influence high-risk behaviors with drugs other than opiates, such as ecstasy or tobacco [20,21]. And in two studies examining the awareness of condom use to prevent the spread of HIV among non-injection drug using based samples (hotel workers in Madrid, adolescents in the United States), both showed that belief in the effectiveness of condoms contributed to more likely use [22,23]. The effectiveness of condom use education and provision in reducing the risk of HIV transmission has been widely supported in the research literature among various populations [24,25].

The international literature by and large supports the effectiveness of harm reduction programming in prison settings [26]. The benefit of needle exchange programs, for example, in the reduction of risk behaviour and the transmission of blood-borne infection in correctional facilities in such places as Germany, Spain and Switzerland has been supported through research [27]. More specific, the importance of informing prisoners about the effectiveness of harm reduction initiatives for changing their HIV high-risk behavior has received some support in this study, and as reviewed, in others as well. Considering these findings and our understanding of the prison environment, prisoners need to be viewed as individuals who are capable of making health informed choices, and not simply criminals who are incarcerated to be punished [28,29]. This damaging ideology is one of many barriers globally that must be overcome if a harm reduction approach, in particular among a prison population, is to be fully embraced and implemented. In Iran, triangular clinics are suggested to be a very viable and possible step toward ensuring this.

The integrated concept of triangular clinics (sexually transmitted infections, HIV/AIDS, drug abuse) in Iran prisons, including Rajaei Shahr prison, attempt to reduce the threat of HIV transmission that prisoners face while incarcerated. Triangular clinics are well-established complex clinics serving a wide range of prisoner health needs, including counseling and testing, harm reduction interventions (e.g., needle exchange) and medical diagnosis and treatment for sexually transmitted infections [30]. According to the findings of this study, to improve the value of these services, it may be wise to widely educate the prison population about their effectiveness. Once again, this suggests that it is necessary that prisoners be viewed as individuals with the capacity and desire to make informed decisions about their own health.

## Conclusion

For many reasons, Iran has a large and growing prison population. Of great concern is the high rate of HIV/AIDS among prisoners, and the need to stop transmission of the disease. Within the Iranian prison environment, it is most commonly spread through injection drug use and sexual contact. This is similar to the global situation [1,2,31,32].

According to the results of this study, HIV high-risk behaviors are common among a sample of adult males incarcerated at the Rajaei-Shahr prison in Iran, despite the fact that they are well informed about the potential for HIV transmission. For risky behaviors related to sexuality, this may be due to the fact that sexual behavior is still a taboo in Iran and is not openly discussed. For example, in our study it is where there was the highest misperception about HIV, that is, that it can be transferred through shaking hands and kissing with an HIV infected individual (95% incorrectly reported this). Consequently, misconceptions prevail and individuals may be unwilling to seek services within the prison system. For various reasons, including stigma, prisoners also are known to be hesitant to access related harm-reduction services for their injection drug use. This study suggests the need to educate prisoners on the effectiveness of harm reduction measures for all HIV-related risky behaviours, as it may lead to a reduction in high-risk behaviours. Given that Iran is progressive in its offering of services, this is even more important.

As mentioned, a limitation of our research methodology is our use of a cross-sectional design did not allow us to identify any causal relationship between the cause (prisoners' beliefs) and the outcome (prisoners' behaviours). An important next step in this research is to conduct multivariate analyses to permit some statistical control of important factors.

We suggest that work in the area of education needs to ensure that a cultural approach that accounts for the religious and social norms of Iran be explored [33]. For example, according to Islamic belief any activity which endangers an individual's life is strictly prohibited (e.g., illicit drug use). As we know from other work, education efforts must also address the mistrust between prisoners and correctional administration, as well as low levels of prisoner literacy [1]. We suggest as well the need to explore various venues to provide education on the effectiveness of HIV harm reduction interventions. For example, consideration could be given to having peer educators, counselors, support groups, targeting specific populations, creating videos and offering drama lessons to address the knowledge, attitudes and behaviours toward HIV/AIDS among prisoners. An important role in such initiatives is having prisoners participate in their design to ensure that specific populations receive the information in the most valuable and applicable way possible. Such initiatives have been implemented elsewhere with success [34,1]. Knowledge assessment studies would be beneficial for monitoring the effectiveness of such training and educational efforts.

And finally, it is important that the findings of this study be considered beyond the walls of the prison environment, because the majority of HIV infected prisoners will be released from prison and will reintegrate into the general Iranian population and potentially contribute to the spread of HIV in their home communities. AIDS awareness still remains limited among many sectors of Iranian society, including the wives and partners of ex-prisoners. Censorship exists in some sectors of society and HIV/AIDS is still highly stigmatized as a social taboo. It follows that the prison education efforts suggested in this study need to be part of a strong, comprehensive and large-scale HIV/AIDS education and communication strategy in Iran.

## Competing interests

The authors declare that they have no competing interests.

## Authors' contributions

BE and RTA participated in the design of the study, collection of data, analysis and interpretation of data, drafting the article, and final approval of this version, CAD participated in contextualizing the data, drafting the article, and final approval of this version, PM, MK and JW reviewed and suggested revisions on the research methodology and the approach to data analysis and presentation of the results, and final approval of this version.

## References

[B1] Kantor E, Peiperl L, Coffey S, Bacon O, Volberding P (2006). HIV transmission and prevention in prisons. HIV InSite Knowledge Base Chapter.

[B2] Krebs CP, Simmons M (2002). Intraprison HIV transmission: An Assessment of Whether it Occurs, How it Occurs, and Who is at Risk. AIDS Educ Prev.

[B3] Salazar C, Hamidreza S (2006). Uniting the World Against AIDS Iran (Islamic Republic of).

[B4] Anonymous Expert HIV/AIDS Counselling IRAN: HIV/AIDS and intravenous drug usage.

[B5] Ohiri K, Claeson M, Rassaghi E, Nassirimanesh B, Afshar P, Power R HIV/AIDS Prevention among Injection Drug Users: Learning from Harm Reduction in Iran.

[B6] Single E (1995). Defining harm reduction. Drug Alcohol Rev.

[B7] Thomas G (2005). Harm Reduction Policies and Programs for Persons Involved in the Criminal Justice System.

[B8] Zamani S, Kihara M, Gouya MM, Vazirian M, Ono-Kihara M, Razzaghi EM, Ichikawa S (2005). Prevalence of factors associated with HIV-1 infection among drug users visiting treatment centres in Tehran, Iran. AIDS.

[B9] Becker MH, Radius SM, Rosenstock IM (1978). Compliance with a medical regimen for asthma: a test of the health belief model. Public Health Reports.

[B10] Glanz K, Rimer BK, Lewis FM (2002). Health Behavior and Health Education Theory, Research and Practice.

[B11] Matsuo EUH (2003). Impact of Knowledge, Attitude, and Beliefs about AIDS on Sexual Behavioral Change among College Students in Nigeria: The Case of the University of Nigeria Nsukka.

[B12] Silva IDS, Silva, IDS (1999). Cross-Sectional Surveys. Cancer Epidemiology: Principles and Methods.

[B13] Nakhaee FH (2002). Prisoners knowledge of HIV/AIDS and its prevention in Kerman, Islamic Republic of Iran. Eastern Mediterranean Health Journal.

[B14] Ebadifard Azar FFM, Hedayat Rad M, Mousavyan Poor MK (2003). Evaluating high school HIV/AIDS education: Implications of intervention. Hakim Research Journal.

[B15] Smith Fawzi MC, Jagannathan P, Cabral J, Banares R, Salazar J, Farmer P, Behforouz H (2006). Limitations in knowledge of HIV transmission among HIV-positive patients accessing case management services in a resource-poor setting. AIDS Care.

[B16] Odujinrin MT, Adebajo SB (2001). Social characteristics, HIV/AIDS knowledge, preventive practices and risk factors elicitation among prisoners in Lagos, Nigeria. West African Journal of Medicine.

[B17] Tavoosi A, Zaferani A, Enzevaei A, Tajik P, Ahmadinezhad Z (2004). Knowledge and attitudes towards HIV/AIDS among Iranian students. BMC Public Health.

[B18] Burrows D (2006). Advocacy and coverage of needle exchange programs: Results of a comparative study of harm reduction programs in Brazil, Bangladesh, Belarus, Ukraine, Russian Federation, and China. Cad Saude Publica.

[B19] Rose VJ, Raymond HF, Kellogg TA, McFarland W (2006). Assessing the feasibility of harm reduction services for MSM: The late night breakfast buffet study. Harm Reduction Journal.

[B20] Allott K, Redman J (2006). Patterns of use and harm reduction practices of ecstasy users in Australia. Drug Alcohol Depend.

[B21] Savitz DA, Meyer RE, Tanzer JM, Mirvish SS, Lewin F (2006). Public health implications of smokeless tobacco use as a harm reduction strategy. American Journal of Public Health.

[B22] Kumar S (1993). KAB study on AIDS awareness among hotel employees. International AIDS Conference.

[B23] Hingson R, Strunin L (1989). Do health belief model beliefs about HIV infection and condoms predict adolescent condom use?. International AIDS Conference.

[B24] Rekart ML (2005). Sex-work Harm Reduction. Lancet.

[B25] Prata N, Morris L, Mazive E, Vahidnia F, Stehr M (2006). Reationship between HIV risk perception and condom use: Evidence from a population-based survey in Mozambique. International Family Planning Perspectives.

[B26] Rehm J, Betteridge G, Stöver H, Laticevschi D, Nelles J (2004). Prison Needle Exchange: Lessons from a Comprehensive Review of the International Evidence and Experience.

[B27] Dolan K, Rutter S, Wodak AD (2003). Prison-based syringe exchange programmes: A review of international research and development. Addiction.

[B28] Kongsakon R, Pocham N (2006). Legal harm reduction among intravenous drug users. Journal of the Medical Association of Thailand.

[B29] (1993). WHO Guidelines on HIV Infection and AIDS in Prisons.

[B30] (2004). Best Practice in HIV/AIDS Prevention and Care for Injecting Drug Abusers: The Triangular Clinic in Kermanshah, Islamic Republic of Iran.

[B31] Wood E, Li K, Small W, Montaner JS, Schechter MT, Kerr T (2005). Recent incarceration independently associated with syringe sharing by injection drug users. Public Health Reports.

[B32] Brewer TF, Vlahov D, Taylor E, Hall D, Munoz A, Polk BF (1988). Transmission of HIV-1 within a statewide prison system. AIDS.

[B33] Hasnain M (2005). Cultual Approach to HIV/AIDS Harm Reduction in Muslim Countries. Harm Reduction Journal.

[B34] Botswana Institute for Development Policy Analysis (2005). Study on knowledge, attitude and behaviour toward HIV/AIDS in the vocational training sector.

